# Non-invasive monitoring of blood pressure using the Philips Intellivue MP50 monitor cannot replace invasive blood pressure techniques in surgery patients under general anesthesia

**DOI:** 10.3892/etm.2013.1121

**Published:** 2013-05-17

**Authors:** XIANGHU MENG, GUANGHUI ZANG, LONGCHANG FAN, LEI ZHENG, JINZHEN DAI, XUEREN WANG, WEI XIA, JIHONG LIU, CHUANHAN ZHANG

**Affiliations:** 1 Departments of Urology, Huazhong University of Science and Technology, Wuhan, Hubei 430030;; 2 Anesthesiology, Tongji Hospital, Tongji Medical College, Huazhong University of Science and Technology, Wuhan, Hubei 430030;; 3 Anhui Institute of Geriatrics, Ma Anshan, Anhui 243000, P.R. China

**Keywords:** intra-arterial blood pressure, oscillometric blood pressure, correlation, Bland-Altman, agreement

## Abstract

The Philips Intellivue MP50 monitor provides a method for non-invasive, near-continuous blood pressure (BP) monitoring and is designed to be an alternative to direct intra-arterial BP (IABP) measurement. However, no studies have specifically compared non-invasive and invasive BP measurements using the monitor. The present retrospective study observed 515 patients undergoing surgery with general anesthesia, whose invasive (intra-radial, femoral or dorsalis pedis artery) and non-invasive (oscillometric) BP (NIBP) were monitored simultaneously using the monitor. These data were analyzed using correlations, regressions and Bland-Altman plots. The patients were placed in a supine position during surgery. The correlation data for invasive BP and NIBP measurements were: for intra-radial measurements, r^2^=0.51 (bias and precision, 11.04±15.22 and 14.76±11.64 mmHg, respectively) for systolic BP (SBP) and r^2^=0.27 (6.17±11.95 and 9.77±9.25 mmHg, respectively) for diastolic BP (DBP); for intra-femoral measurements: r^2^=0.57 (14.79±14.55 and 17.15±11.68 mmHg, respectively) for SBP and r^2^=0.45 (4.12±9.70 and 7.49±7.40 mmHg, respectively) for DBP; and for intra-dorsalis pedis measurements: r^2^=0.33 (13.00±16.81 and 17.34±12.28 mmHg, respectively) for SBP and r^2^=0.30 (0.17±11.27 and 8.44±7.46 mmHg, respectively) for DBP. According to this data, the NIBP measured by the Philips Intellivue MP50 monitor showed low positive correlations and poor agreement with the IABP, as calculated by Bland-Altman analysis. Therefore, the use of oscillometric BP measured by the monitor in surgery patients under general anesthesia is not generally recommended.

## Introduction

It is essential for clinicians to monitor the arterial blood pressure (BP) of surgery patients who are under general anesthesia. BP is a basic vital sign and is one of the most important hemodynamic indices often utilized to guide therapeutic interventions, particularly as part of the standard of care for anesthesia and perioperative management. Acquiring timely and accurate BP information is critical for monitoring the depth of anesthesia and for guaranteeing the safety of the patient while operating under general anesthesia. Inaccurate measurements of BP may lead to inappropriate interventions.

BP may be measured by either invasive or non-invasive methods in anesthetized patients. Intra-radial, intra-femoral and intra-dorsalis pedis artery BP measurements are the most common invasive methods used. By contrast, oscillometric BP measurement is the most common non-invasive method used during surgery. Invasive and non-invasive methods each have their own advantages and disadvantages. Traditionally, BP measurements taken via invasive methods are considered the gold standard and most accurately reflect the BP at any given time (1). However, the placement of an arterial catheter in patients is often technically challenging, costly and accompanied by several complications, including trauma, bleeding, infection, thrombosis, embolism, distal ischemia and the formation of pseudoaneurysms (2–5). Although non-invasive methods are most commonly used in routine surgeries due to convenience, non-invasive BP (NIBP) measurements are less accurate and may be impacted by a number of factors.

The comparison of BP measurements by invasive and non-invasive sphygmomanometry has been a topic of study for decades. Nevertheless, the extent of agreement between invasive and non-invasive monitoring devices remains unknown. For instance, in studies that compared NIBP monitoring techniques performed using the Philips MP90 (Philips Medical Systems, BG Eindhoven, The Netherlands), Nexfin HD (BMEYE, Amsterdam, The Netherlands) and Finapres units (Finapres Medical Systems, Amsterdam, The Netherlands) with those using intra-arterial BP (IABP) monitoring, inconsistent data were observed (6–9). However, NIBP data collected using the T-Line Tensymeter (Tensys Medical, Inc., San Diego, CA, USA), Colin CBM-3000 (Colin Electronics, Komaki, Japan) and Vasotrac (Medwave, Arden Hills, MN, USA) instruments displayed good agreement with the intra-radial BP (10–16). Although marked agreement was observed between the Vasotrac and intra-radial artery BP, various studies have identified differing biases affecting the systolic BP (SBP), diastolic BP (DBP) and mean arterial blood pressure (MAP) measurements (12–16). Several studies have shown that these invasive and non-invasive methods produce different values. The variations are likely to be due to differences between the devices, the positions and cuff selections for the non-invasive measurements, the age, weight and surgical status of the patients, the range of narcotics administered and the varying patient positions for surgery (17–21). However, to the best of our knowledge, no studies have specifically compared non-invasive and invasive BP measurements using the Philips Intellivue MP50 monitor in surgery patients under general anesthesia.

Therefore, the present retrospective study included 515 cases where the BP had been monitored using invasive methods at the intra-radial, femoral or dorsalis pedis arteries and oscillometric methods using the Philips Intellivue MP50 monitor. All cases involved surgeries performed under general anesthesia between January 2009 and April 2011 in Tongji Hospital (Hubei, China). The aim of the study was to identify the correlation and agreement between the standard invasive method and the oscillometric method using the Philips Intellivue MP50 monitor.

## Patients and methods

### 

#### Participants

A retrospective review was performed of patients who had undergone surgeries in which their BP was monitored by invasive and non-invasive methods. The patients had been treated at Tongji Hospital between January 2009 and April 2011. In total, the data of 515 patients were retrieved from files that were kept in the Department of Anesthesia. The patients were of American Society of Anesthesiologists (ASA) classification 1 or 2. Patients suffering from cardiovascular disease, high BP or diabetes were excluded from the study.

### Procedures and outcomes

#### Measurement of NIBP

Patients were transferred to the operating room in the supine position, where they were attached to standard monitors, including an electrocardiography machine, an SpO_2_ monitor and a sphygmobolometer. Following a 10 min stabilization period, the NIBP (oscillometric method) from the humerus of the right arm was measured using the Philips Intellivue MP50 monitor.

#### Measurement of IABP

All 515 patients were of ASA physical status 1 or 2 and were scheduled to undergo elective surgery at Tongji Hospital. All surgeries were performed under general anesthesia in the central operating room area and were prospectively entered into the present study. Entropy electrodes were applied to the right forehead of each patient and a Narcotrend monitor (MonitorTechnik, Bad Bramstedt, Germany) was used to detect whether the depth of anesthesia became insufficient at any time during the study.

The general anesthesia procedure was as follows: Tidal volume method inhalation induction (8% sevoflurane oxygen flow 6 l/min) followed by intravenous injections of femifentanil (1 *μ*g/kg) and rocuronium (0.6 mg/kg). Tracheal intubation was provided at a train of four (TOF) stimulation value of 0. Fentanyl (2 *μ*g/kg) was injected 3 min prior to the incision and the anesthesia was maintained with remifentanil (0.3 *μ*g/kg/min) and sevoflurane to keep the Narcotrend value between 20 and 46. The end tidal concentration of sevoflurane was monitored continuously.

Following the induction of anesthesia, the same primary team placed a 20-gauge catheter in the radial artery of the wrist, the femoral artery of the inguinal region or the dorsalis pedis artery of the instep. The location of the IABP was determined by the disease and surgical site of the patient. The arterial catheter was connected to a disposable pressure transducer (Edwards Life Sciences, Irvine, CA, USA), which was calibrated to the level of the patient’s heart. The tubing and the transducer were inspected to ensure that there were no technical issues or air bubbles that may have caused an erroneous recording. The Philips Intellivue MP50 monitor was then interfaced with the patient to allow simultaneous IABP data collection.

Once the hemodynamic changes had stabilized and the preparations were complete, the invasive and non-invasive BP were measured simultaneously and recorded every 5 min. To minimise the errors caused by movement and reference point changes, only patients who were not moved during surgery were selected for the study.

#### Ethics

Approval for the present study was obtained from The Ethics Committee of Tongji Medical College, Huazhong University of Science and Technology (China). In accordance with this approval for a retrospective analysis of patient data, no individual patient consent was required.

#### Statistical analysis

The data were analyzed using SPSS software (version 12.0; SPSS, Inc., Chicago, IL, USA). Results are presented as the mean ± standard deviation. The various invasive BP measurements and oscillometric methods were examined using correlation, regression and Bland-Altman analyses (22). P<0.05 was considered to indicate a statistically significant difference.

## Results

### 

#### Baseline characteristics and clinical data

The clinical characteristics of the study population are summarized in Table I. A total of 515 patients that were scheduled to undergo elective surgery were enrolled in the present retrospective study: The intra-radial group consisted of 165 patients (85 males and 80 females; mean age, 55±16 years; mean weight, 62±12 kg). The intra-femoral group consisted of 179 patients (96 males and 83 females; mean age, 57±14 years; mean weight, 58±11 kg). A total of 171 patients (86 males and 85 females; mean age, 42±16 years; mean weight, 59±12 kg) formed the intra-dorsalis pedis artery group.

#### Comparison between the intra-radial and oscillometric BP

**Figure 1.**
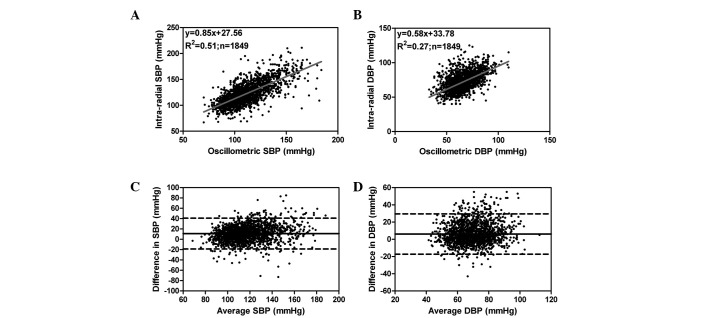
Comparison between intra-radial and oscillometric blood pressure (BP) measurements. Correlations between (A) SBP and (B) DBP estimated using the intra-radial and oscillometric methods. Bland-Altman plots of the same data for (C) SBP and (D) DBP. Solid line, mean; dashed lines, ±1.96 standard deviation; SBP, systolic blood pressure; DBP diastolic blood pressure.

The correlation and the regression and Bland-Altman analyses of SBP and DBP between the intra-radial and oscillometric BP measurements are shown in Fig. 1 and Table II. Based on 1,849 measurements from 165 patients, there was a moderate correlation between the intra-radial and oscillometric measurements for SBP (r^2^=0.51, P<0.001) and a limited correlation for DBP (r^2^=0.27, P<0.001). The Bland-Altman analysis showed poor agreement for the SBP (mean bias of 11.04±15.22, with a precision of 14.76±11.64 mmHg) and DBP (mean bias of 6.17±11.95, with precision of 9.77±9.25 mmHg), measured using the intra-radial and oscillometric methods, with limits of agreement ranging from 40.87 to −18.79 mmHg and 29.59 to −17.25 mmHg, respectively. Between the methods, 38.78% of the SBP values and 38.34% of the DBP values differed by >10 mmHg.

#### Comparison between the intra-femoral and oscillometric BP

**Figure 2.**
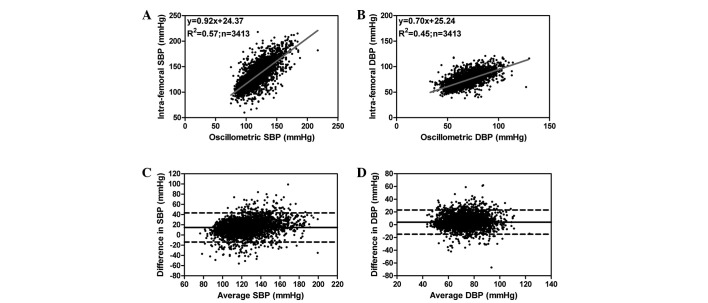
Comparison between the intra-femoral and oscillometric blood pressure (BP) measurements. Correlations between (A) SBP and (B) DBP estimated using the intra-femoral and oscillometric methods. Bland-Altman plots of the same data for (C) SBP and (D) DBP. Solid line, mean; dashed lines, ±1.96 standard deviation; SBP, systolic BP; DBP, diastolic BP.

The correlation and the regression and Bland-Altman analyses of the SBP and DBP between the intra-femoral and oscillometric BP measurements are shown in Fig. 2 and Table II. Based on 3,413 measurements from 179 patients, there was a moderate correlation between the intra-femoral and oscillometric measurements for the SBP (r^2^=0.57, P<0.001) and a limited correlation for the DBP (r^2^=0.45, P<0.001). The Bland-Altman analysis showed poor agreement for the SBP (mean bias of 14.79±14.55 with precision of 17.15±11.68 mmHg) and the DBP (mean bias of 4.12±9.70 with precision of 7.49±7.40 mmHg) measured using intra-femoral and oscillometric methods, with limits of agreement ranging from 43.31 to −13.73 mmHg and 23.13 to −14.89 mmHg, respectively. Between the methods, 72.25% of the SBP values and 27.92% of the DBP values differed by >10 mmHg.

#### Comparison between the intra-dorsalis pedis and oscillometric blood pressure

**Figure 3.**
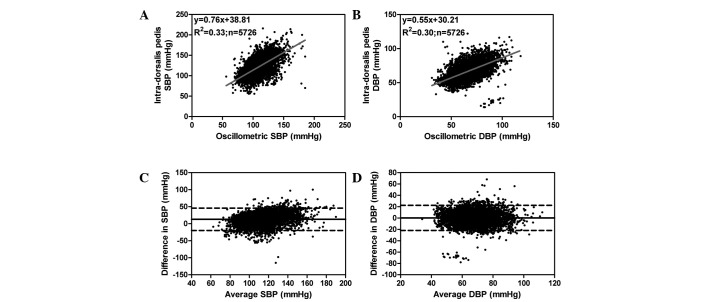
Comparison between the intra-dorsalis pedis and oscillometric blood pressure (BP) measurements. Correlations between (A) SBP and (B) DBP estimated using intra-dorsalis pedis and oscillometric method. Bland-Altman plot of the same data for (C) SBP and (D) DBP. Solid line, mean; dashed lines, ±1.96 standard deviation; SBP; systolic BP; DBP, diastolic BP.

The correlation and the regression and Bland-Altman analyses of the SBP and DBP are shown in Fig. 3 and Table II. Based on 5,726 measurements from 171 patients, there were limited correlations between the intra-dorsalis pedis and oscillometric measurements for SBP (r^2^=0.33, P<0.001) and DBP (r^2^=0.30, P<0.001). The Bland-Altman analysis showed poor agreement for SBP (mean bias of 13.00±16.81, with precision of 17.34±12.28 mmHg) and DBP (mean bias of 0.17±11.27, with precision of 8.44±7.46 mmHg) measured using intra-dorsalis pedis and oscillometric methods, with limits of agreement ranging from 45.95 to −19.95 mmHg and 22.26 to −21.92 mmHg, respectively. Between the methods, 69.44% of the SBP values and 34.46% of the DBP values differed by >10 mmHg.

## Discussion

The accurate measurement of BP is essential for the rational hemodynamic management of surgery patients. However, it is unclear whether invasive and non-invasive BP measurements may be used interchangeably. The data from the present study revealed that the non-invasive method using the Philips Intellivue MP50 monitor was not an appropriate substitute for standard invasive BP measurement techniques, including those for intra-radial, intra-femoral and intra-dorsalis pedis artery blood pressure, thus supporting the use of direct intra-arterial methods for monitoring BP and guiding treatment decisions due to the accuracy of the invasive methods.

BP is the pressure exerted by circulating blood upon the walls of the blood vessels (23). Invasive and non-invasive techniques reflect the effects of all fluids. However, the two techniques have intrinsic differences as they involve the measurement of different quantities. For example, in the invasive technique, the sum of the lateral pressure (measured by the non-invasive BP) and the converted kinetic energy are recorded. Accordingly, the invasive and non-invasive methods of measurement are different (24,25). Moreover, differences between non-invasive and invasive BP measurements have been documented in various clinical situations (6–21).

The Philips Intellivue MP50 monitor provides a non-invasive, near-continuous method for monitoring BP, and is designed to be an alternative to direct IABP measurement. By compressing the artery with a cuff and then slowly releasing the pressure, pulsations from the artery are transmitted as oscillations to the cuff and the SBP and DBP values are recorded. In the Tongji hospital, the Philips Intellivue MP50 monitor is used to monitor the NIBP and IABP of surgery patients in the operating room. However, there is little information with regard to the correlation and agreement between the NIBP measured by the Philips Intellivue MP50 and the IABP in surgery patients under general anesthesia.

In the present study, measurements from various IABP locations (intra-radial, intra-femoral and intra-dorsalis pedis arteries) were compared with measurements obtained by the oscillometric method from the humerus of the right arm of surgery patients under anesthesia in the supine position. It was identified that there were clinically low positive correlations and poor agreement between the direct BP measurements, including the intra-radial, intra-femoral and intra-dorsalis pedis BP measurements, and the oscillometric BP for the SBP and DBP measured by the Philips Intellivue MP50. The data demonstrated that the mean bias and precision of the DBP between the intra-femoral BP and the NIBP were within the minimum performance standards set by the Association for the Advancement of Medical Instrumentation (AAMI), which recommended that non-invasive BP devices should be accurate within 5 mmHg and have a precision within 8 mmHg. However, the results also demonstrated that between all methods, >10% of the arterial BP values of the SBP and DBP differed by >10 mmHg, which was not in agreement with the standards proposed by the AAMI (26). In addition, the process of making clinical and therapeutic decisions was weakened by the calculated standard deviations of ±15.22, ±11.95, ±14.55, ±9.70, ±16.81 and ±11.27 mmHg, which were determined for the oscillometric method.

A possible explanation for the observed differences between the two systems may be that since the oscillometric method is not standardized, algorithm measurements may differ between manufacturers and even between devices. Belani *et al* (12), observed a good correlation and agreement between the IABP measurements and the Vasotrac (a device that uses frequent gentle compression and decompression of the radial artery at the wrist and displays the arterial pressure wave approximately every 12 to 15 heart beats) in a study of 80 critically ill surgical patients positioned in the supine position. The study demonstrated a bias and precision of 0.0±5.4 and 3.9±3.7 mmHg, respectively, for the SBP and −0.4±3.9 and 2.7±2.8 mmHg, respectively, for the DBP. Moreover, in another study by Belani (13), the differences between the measurements did not exceed 10 mmHg for >90% of the paired values. McCann *et al* (15) reached the same conclusion by comparing the radial artery BP determined by the Vasotrac device and IABP monitoring in adolescents undergoing scoliosis surgery. Janelle and Gravenstein (10) identified a good agreement between the T-Line Tensymeter (continuous non-invasive blood pressure management device) BP and IABP measurements in surgery patients, with a bias and precision of 1.7±7.0 and 5.7±4.4 mmHg, respectively, for the SBP, 2.3±6.9 and 5.7±4.5 mmHg, respectively, for the DBP and 1.7±5.3 and 4.0±4.8 mmHg, respectively, for the mean BP.

Another explanation for the observed difference may be due to the anesthesia. Lakhal *et al* (27) reported that the IABP and arm oscillometric non-invasive MAP readings of patients without general anesthesia were significantly and positively correlated (r^2^=0.85; P<0.001) and that the agreement between these two methods was acceptable (mean bias, 3.4±5.0 mmHg; lower/upper limit of agreement, −6.3/13.1 mmHg). In addition to this, Lee *et al* (18) observed that the values of arterial BP measurements were as high as those measured by non-invasive methods under general anesthesia using sevoflurane. This is in contrast to the results from the present study. However, Lee *et al* did not perform a correlation and agreement analysis between the IABP and NIBP.

Certain limitations of the present study require discussion. Firstly, the NIBP of the 515 patients was recorded from the humerus of the right arm. There are no comparisons between the NIBP or the IABP of the thigh or ankle. One reason for this is that in the majority of hospitals, the NIBP of patients was recorded from the arm due to its practicality and simplicity. Recordings were taken from the thigh or ankle in only a few patients for specific reasons, e.g., due to arm diseases. Another reason is that the NIBP of the arm, measured oscillometrically, has a relatively good agreement with the IABP, whereas the NIBP for the thigh and ankle using the invasive reference shows less agreement (27). Thus, in the present study comparing NIBP with IABP, the NIBP measurements were obtained from the arm rather than the thigh or ankle. Secondly, the focus of the study was on the SBP and DBP, rather than on analyzing the accuracy of oscillometric blood pressure measurements of the MAP. The SBP and DBP are values that are directly measured by the Philips Intellivue MP50 monitor system. By contrast, the MAP is deduced from the SBP and DBP. Therefore, the SBP and DBP values were used as direct measurements of BP values, rather than the calculated value of the MAP.

Although widely used, the oscillometric method of measuring blood pressure used by the Philips Intellivue MP50 monitor was inaccurate in this subset of surgery patients under general anesthesia, and the parameters obtained should be used cautiously. Therefore, the results from the present study suggest that the use of the oscillometric method monitoring system in surgery patients under general anesthesia should not be generally recommended. Whether such a tool may be reliable in certain other patients remains to be determined.

## Figures and Tables

**Table I.** Patient characteristics.

Factors	Location of IABP monitoring		
	Intra-radial artery	Intra-femoral artery	Intra-dorsalis pedis artery
Male	85	96	86
Female	80	83	85
Age (years)	55±16	57±14	42±16
Weight (kg)	62±12	58±11	59±12
Head and neck surgery (n)	-	-	160
Chest surgery (n)	59	82	6
Abdominal surgery (n)	83	97	5
Pelvic surgery (n)	19	-	-
Limb surgery (n)	4	-	-
Number of patients	165	179	171

Age and weight values are expressed as mean ± standard deviation. IABP, intra-arterial blood pressure.

**Table II.** Bias, limits of agreement and precision between IABP and NIBP for SBP and DBP.

Group	Mean bias (mmHg)	Upper/lower limit of agreement (mmHg)	Precision (mmHg)	Measurements, n
Intra-radial BP				
SBP	11.04±15.22	40.87/−18.79	14.76±11.64	1849
DBP	6.17±11.95	29.59/−17.25	9.77±9.25	1849
Intra-femoral BP				
SBP	14.79±14.55	43.31/−13.73	17.15±11.68	3413
DBP	4.12±9.70	23.13/−14.89	7.49±7.40	3413
Intra-dorsalis pedis BP				
SBP	13.00±16.81	45.95/−19.95	17.34±12.28	5726
DBP	0.17±11.27	22.26/−21.92	8.44±7.46	5726

Bias and precision values are expressed as mean ± standard deviation. Bias for each pair of measurements was calculated using IABP-NIBP, and precision was calculated using the absolute difference. BP, blood pressure; IABP, intra-arterial BP; NIBP, non-invasive BP; SBP, systolic BP; DBP, diastolic BP.
